# Cyanidin-3-o-glucoside (C3G) inhibits vascular leakage regulated by microglial activation in early diabetic retinopathy and neovascularization in advanced diabetic retinopathy

**DOI:** 10.1080/21655979.2021.1996512

**Published:** 2021-11-24

**Authors:** Fangling Zhao, Xiang Gao, XiaoJuan Ge, Jiawen Cui, Xia Liu

**Affiliations:** a Faculty of Medicine, Nantong University Medical School, Nantong, China; b School of Life Science, Nantong University, Nantong, China; c Gynaecology and Obstetrics, Zhongshan Hospital of Fudan University, Shanghai, China

**Keywords:** Cyanidin-3-o-glucoside (C3G), diabetic retinopathy (DR), microglial activation, neovascularization

## Abstract

Cyanidin-3-O-glucoside (C3G) is a kind of anthocyanin which shows strong anti-inflammation, anti-tumor and anti-oxidant properties. This paper was designed to explore the potential effects of C3G on diabetic retinopathy (DR). C57BL/6 mice were administrated with streptozotocin (STZ) or vehicle control for the establishment of diabetic models. To simulate hyperglycemia and hypoxia, D-glucose (30 mM) and CoCl_2_ (200 μm/l) were utilized to treat HRECs, respectively. The migration, invasion, inflammation and tube formation abilities of cells were evaluated with the adoption of wound healing, transwell, ELISA and tube formation assays, respectively. Besides, immunofluorescence staining was utilized to detect proliferation and retinal vessels. Evans blue permeation assay were performed to evaluate the vascular leakage in DR mice. Moreover, western blot and qPCR were used to quantify the mRNA and protein expressions of ionized calcium-binding adapter molecule (Iba)-1 and tight junction proteins. Results showed that C3G alleviated the inflammation, microglial activation and angiogenesis in DR mice. Moreover, the proliferation and inflammation of BV2 cells induced by high glucose (HG) were suppressed by C3G. Evans blue permeation assay demonstrated the potency of C3G in attenuating vascular leakage. In addition, C3G suppressed the migration, invasion and angiogenesis of human retinal endothelial cells (HRECs) DR model in vitro.

By confirming the role of C3G in inhibiting vascular leakage regulated by microglia activation in early DR and angiogenesis in advanced DR, this study pointed out the potential of C3G as a therapeutic drug for DR management.

## Introduction

Diabetic retinopathy (DR) is a microvascular complication of diabetes and the research of its mechanism could help develop new therapeutic drugs [1–3]. It remains a major cause of vision loss among people aged 20 to 74 in the world [4]. The occurrence and progression of DR can be resulted from many factors, including renal disease, uncontrolled blood pressure, poor glucose control, elevated glycosylated hemoglobin, and duration of disease [5]. DR can be grouped into non-proliferative retinopathy (NPDR) and proliferative DR (PDR), which respectively indicates the earliest and the advanced phase of DR [6,7]. NPDR features typically asymptomatic microvascular changes while PDR involves angiogenesis [6]. In present, the treatment of DR mainly adopts Scatter or panretinal laser photocoagulation (PRP) [8,9]. However, the use of PRP would lead to the impairment of retinal tissue, thereby causing some symptoms, such as decreased contrast sensitivity and peripheral visual field defects [10]. Furthermore, due to the complex pathology of DR, the current demands for identifying new targets or drugs for the treatment of DR have not been met.

The characterization of DR includes abnormal growth and leakage of small blood vessels, which lead to local edema and dysfunction of related tissues. The dysregulation of vascular regeneration and inflammation are considered to involve in in pathological mechanism DR [11,12]. Cyanidin-3-O-glucoside (C3G) is a kind of anthocyanin which shows strong anti-inflammation, anti-tumor and anti-oxidant properties [13–15]. It is reported that C3G could improve dysfunction of mitochondria [16]. One previous research has shown that C3G, an important player in diabetes, can alleviate diabetic cataract. and diabetic cardiomyopathy [17,18]. In addition, the role of C3G in inhibiting VEGF and angiogenesis was also testified [19,20]. Inflammation and angiogenesis play an important role in DR. Moreover, the protective effects of C3G in 4-hydroxynonenal and visible light-induced retinal injury have been reported [21].

In this paper, it is assumed that C3G can inhibit retinal inflammatory injury in the early stage of DR and angiogenesis in the late stage of DR. To clearly reveal the intricate mechanism of DR, and the impact of C3G on the progression of DR, we used the widely acknowledged streptozotocin (STZ) model for the simulation of diabetic mice model.

## Materials and methods

### Establishment of NPDR and PDR mice model induced by streptozocin (STZ)

The C57BL/6 mice were purchased from Beijing HFK Bioscience Co. Ltd. (Beijing, China). The mice were fed at 25°C with a 12-h light/dark cycle and felt free to get access to water and standard rodent chow diet. After two weeks of adaptive feeding, to establish the mice model of STZ-induced NPDR, thirty-two C57BL/6 mice weighing 18–22 g were intraperitoneally treated with 55 mg/kg STZ for 5 consecutive days, while the other sixteen mice were treated with normal saline and considered as control group. The concentration of serum glucose was monitored on the 7th day, and mice with glucose concentration over 16.7 mmol/L were taken as diabetic mice. In this experiment, the blood glucose concentration of control mice was normal (n = 16), while diabetic mice were randomly classified into two groups: STZ model (n = 16) and STZ+C3G (POLYPHENOLS AS, Sandnes, Norway. purity > 97%) 20 mg/kg (n = 16) [18]. In STZ+C3G 20 mg/kg group, at 1 month after STZ injection, the mice were administered with C3G (20 mg/kg per day) by gavage for 1 month consecutively.

Thereafter, blood-retinal barrier (BRB) breakdown was conducted by Evans blue in six mice of each group. Other mice were anesthetized by intraperitoneal injection of sodium pentobarbital (30 mg/kg). The blood was collected from abdominal aorta and the retinal tissues were collected immediately.

In order to establish the PDR mice model induced by STZ, C57BL/6 mice were undergone procedures as mentioned above. The mice were grouped into three groups (control, STZ, STZ+C3G 20 mg/kg, each group n = 10). Specifically, 3 months after STZ injection, the rats were gavaged with C3G for 2 months. The mice were anesthetized by intraperitoneal injection of sodium pentobarbital (30 mg/kg). The blood was collected from abdominal aorta and the retinal tissues were collected immediately. The study was approved by the ethical committee of Nantong University Medical School.

### Enzyme-linked immunosorbent assay (ELISA)

The levels of TNF-alpha, IL-1 beta, and IL-6 in the serum and retinas of DR mice were assessed by ELISA kits (KAINOS laboratories, Japan) according to the manufacturer’s protocol. After BV2 cells were treated with HG and C3G for 48 h, the supernatant was collected for the detection of levels of TNF-alpha, IL-1 beta, and IL-6 by ELISA kits (KAINOS laboratories, Japan) according to the manufacturer’s guidance.

### Evans blue leakage assay

Mice were injected with 2% Evans blue (10 µL/g, i.p.) in PBS. After 2 h, mice were sacrificed and perfused with PBS to completely remove the Evans blue dye in blood vessels. Retinas were carefully dissected and the weight was determined after thoroughly drying. Next, the retinas were incubated in 120 µL formamide for 18 h at 70°C to extract Evans blue dye. The extract was centrifuged, and the absorbance of supernatant was measured at 620 nm by a spectrophotometer to determine BRB.

Reverse transcription-polymerase chain reaction (RT-qPCR) Total RNA was extracted from retinas of DR mice and BV-2 or HRECs by Trizol kit (Thermo Fisher Scientific) in accordance with the manufacturer’s procedure. Then, synthesis of cDNA was conducted using a cDNA synthesis kit (Thermo Fisher Scientific). After that, SYBR premix Ex Taq II kit (TaKaRa, Dalian, China) was used to perform RT-qPCR reaction on an ABI 7900HT instrument (ABI, NY, USA). The qPCR reaction conditions were designed as follows: 94°C for 3 min followed by 45 cycles of 94°C for 45s, 56°C for 30s, finally, 72°C for 45s. The primers were obtained from GenePharma (Shanghai, China). Analysis of the data was conducted using 2^−ΔΔCt^method and the levels of mRNA were normalized to GAPDH. The primers sequences are as following: TNF- alpha Forward: 5-’CAGGCGGTGCCTATGTCTC-3ʹ, Reverse: 5ʹ-CGATCACCCCGAAGTTCAGTAG-3ʹ, IL-1 beta Forward: 5ʹ- GAAATGCCACCTTTTGACAGTG −3ʹ, Reverse: 5ʹ- TGGATGCTCTCATCAGGACAG-3ʹ. IL-6 Forward: 5ʹ- CTGCAAGAGACTTCCATCCAG-3ʹ, Reverse: 5ʹ- AGTGGTATAGACAGGTCTGTTGG-3ʹ. Iba-1 Forward: 5ʹ- CTTGAAGCGAATGCTGGAGAA-3ʹ, Reverse: 5ʹ- GGCAGCTCGGAGATAGCTTT-3ʹ. occludin-1 Forward: 5ʹ-CAGCCCTCAGGTGACTGTTATT-3ʹ, Reverse: 5ʹ-AGCCTGGTCTACAGCGTAAGTT-3ʹ. claudin-1 Forward: 5ʹ- TGCCCCAGTGGAAGATTTACT-3ʹ, Reverse: 5ʹ- CTTTGCGAAACGCAGGACAT-3ʹ. ZO-1 Forward: 5ʹ- GCCGCTAAGAGCACAGCAA-3ʹ, Reverse: 5ʹ- GCCCTCCTTTTAACACATCAGA-3ʹ. VEGFForward: 5ʹ- TTTGGCAAATACAACCCTTCAGA-3ʹ, Reverse: 5ʹ- GCTCCAGTATCATTTCCAACCA −3ʹ. VEGFR1 Forward: 5ʹ- CTCAGGGTCGAAGTTAAAAGTGC-3ʹ, Reverse: 5ʹ- TTGCCTGTTATCCCTCCCACA-3ʹ. VEGFR2 Forward: 5ʹ- TTTGGCAAATACAACCCTTCAGA-3ʹ, Reverse: 5ʹ- GCTCCAGTATCATTTCCAACCA-3ʹ. CD31 Forward: 5ʹ- ACGCTGGTGCTCTATGCAAG-3ʹ, Reverse: 5ʹ- TCAGTTGCTGCCCATTCATCA-3ʹ. GAPDH Forward: 5ʹ- AGGTCGGTGTGAACGGATTTG-3ʹ, Reverse: 5ʹ-GGGGTCGTTGATGGCAACA −3ʹ.

### Western blot

The retina of mice and BV-2 or HRECs were collected and homogenized with lysis buffer, followed which centrifugation (1600 × g, 15 min) was performed at 4°C. Protein concentration was quantified in supernatant fluid by bicinchoninic acid assay (Beyotime Institute of Biotechnology, Haimen, China). Separated by sodium dodecyl sulfate polyacrylamide gel electrophoresis (SDS-PAGE), the proteins were transferred to the PVDF membranes (GE Healthcare Europe GmbH, Freiburg, Germany). Proteins were blocked with 5% nonfat milk for 1 h and incubated with primary antibodies (p-p65: ab76302; t-p65: ab32536; occludin: ab216327; claudin-1: ab180158; ZO-1: ab216880; VEGF: ab214424; CD31: ab182981; GAPDH: ab8245. Abcam, England. p-IκB: #2859, t-IκB: #9242, Iba-1: #17,198, Cell Signaling Technology, USA). Subsequently, HRP-conjugated secondary antibody (ab7090, abcam, England) was used to incubate the membranes for 1 h at 37°C. Protein bands were visualized by enhanced chemiluminescence reagent (Millipore Corp., Bedford, MA).

### Immunofluorescent staining

Collected retinal tissues were embedded in paraffin and cut into 5 µm sections. After removing the paraffin, the sections were rehydrated, blocked, and incubated with primary antibodies (anti-Iba-1, ab178846, abcam, England; CD31, AF6191, Affinity, USA) at 4°C overnight. On the second day, after washing with phosphate-buffered saline (PBS), the sections were incubated with secondary antibodies for 30 min at room temperature. Nuclei were counterstained using DAPI for 2 min. BV-2 or HRECs were fixed with 4% paraformaldehyde for 15 min and then treated with 0.1% Triton for 15 min. After washing with PBS for three times, following incubation with 10% normal goat serum for 40 min, BV-2 cells were incubated with primary antibodies at 4°C overnight and then incubated with secondary antibodies at 37°C for 1 h. DAPI was used for staining nuclei for 15 min. Finally, the sections and cells were photographed under a fluorescence microscope (Zeiss).

### Cell culture and treatment

BV2 microglial cells that obtained from ICLC (Genova, Italy) were cultured in DMEM supplemented with 10% FBS, 100 U/mL penicillin, and 100 ✓g/mL streptomycin (Gibco-BRL, Grand Island, NY, USA) at 37°C with 5% CO_2_. The BV2 cells were inoculated in 96-well plates at a density of 2 × 10^4^ cells per well and induced by mannitol (MG;25 mM), high glucose (HG;25 mM) or in combination with 10 µmol/l C3G (Sigma-Aldrich, St. Louis, MO, USA) for 48 h.

HRECs bought from Sciencell (USA, California) were plated in a Series 8000 WJ cell incubator (ThermoScientific, Waltham, MA) in Endothelial Cell Medium (Sciencell, Carlsbad, CA, USA) with 10% FBS, 100 U/ml penicillin, and 100 U/ml streptomycin at an atmosphere of 37°C with 5% CO_2_. To simulate hyperglycemia in DR environment, 30 mM D-glucose was added into HRECs and 200 μM CoCl_2_ was employed to induce hypoxia [22]. The cells were pretreated with C3G 10 μM for 2 h and then treated with D-glucose and CoCl2 for 48 h.

### Wound healing migration assay

After HRECs reached 70–80% confluence, the monolayer of cells was scratched by scraping the plate with a pipette tip. The pictures of wounded monolayers were captured immediately after wounding.

### Transwell invasion assay

HRECs were inoculated in 96-well plates at a density of 2 × 10^4^ cells per well and cultured for 24 h. The noninvasive cells in the upper chamber were removed, while invasive cells embedded in the membrane of the transwell were fixed with 4% paraformaldehyde and stained with crystal violet for 15 min. The cells were observed and counted under a microscope.

### Tube formation assay

Treated HRECs were inoculated in 96-well plates precoated with Matrigel Basement Membrane Matrix (BD Biosciences, Bedford, MA) at a density of 2 × 10^5^ cells/well and incubated for 24 h at 37°C. Images were captured by an inverted phase-contrast microscope (Olympus IX70, Toyko, Japan) and data were processed by ImageJ software (National Institutes of Health).

### Statistical analysis

GraphPad Prism 5 (GraphPad, San Diego, CA, USA) software was used for statistical analysis. One-way ANOVA followed by Tukey’s post hoc test was performed for the comparisons among groups. *P* < 0.05 was considered to show statistically significance.

## Results

The effects of C3G on the inflammation and microglial activation in DR mice

**Figure 1.**
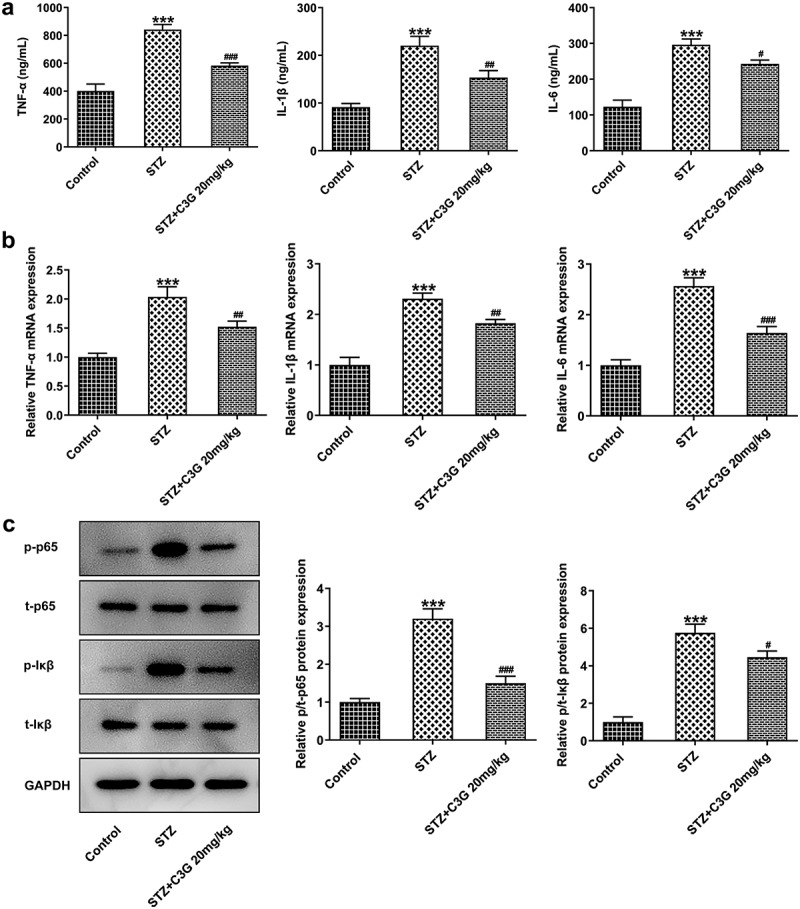
The effects of C3G on the inflammation in DR mice. (a) The release of inflammatory cytokines was detected using ELISA. (b) The mRNA expressions of inflammatory cytokines were detected using RT-qPCR. (c) The protein expressions of p-NF-KB p65 and P-IKβ were measured using western blot. *** *p* < 0.001 vs Control, ^#^ *p* < 0.05, ^##^ *p* < 0.01, ^###^*p* < 0.001 vs STZ+C3G20mg/kg

**Figure 2.**
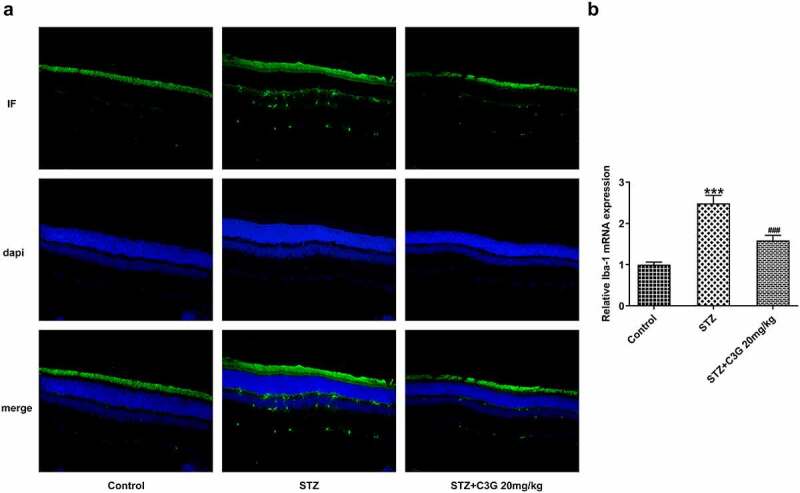
The effects of C3G on the microglial activation in DR mice. (a) Specific staining of microglia was used to mark the effect of glial cell activation in retina. (b) The mRNA expression of Iba-1 detected using RT-qPCR. *** *p* < 0.001 vs Control, ^###^*p* < 0.001 vs STZ+C3G20mg/kg

We wondered to know whether C3G affected inflammation and microglial activation in a NPDR mouse model. Firstly, the inflammation and microglial activation in DR mice were detected. Results obtained from ELISA and RT-qPCR indicated that the expressions of pro-inflammatory cytokines, TNF- alpha, IL-1 beta, and IL-6 induced by STZ was remarkably suppressed by C3G (p < 0.01) (Figure 1a-b). Inflammation-related markers, including p-NF-✓B p65 and p-IK, were measured using western blot. Similarly, the increased expressions of p-NF-✓B p65 and p-IK✓in STZ-induced mice were significantly decreased (p < 0.05) after C3G treatment (Figure 1c). It was also found that the increased expression of microglial cell marker ionized calcium binding adaptor molecule-1 (Iba-1) caused by STZ induction was then significantly reduced by C3G treatment, implying that C3G administration suppressed the microglial activation of DR mice (p < 0.001) (Figure 2a-b). Taken together, C3G alleviated the inflammation and microglial activation in DR mice.

The effects of C3G on the proliferation and inflammation of HG-induced BV2 cells

**Figure 3.**
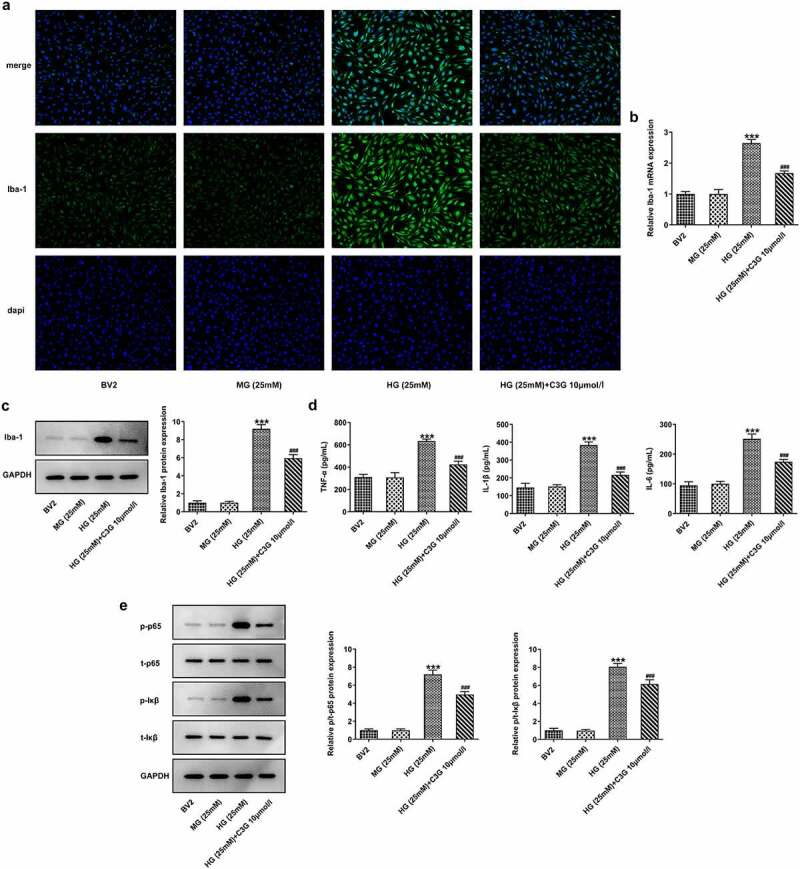
The effects of C3G on the proliferation and inflammation of HG-induced BV2 cells. (a) The number of positive cells was evaluated using immunofluorescence staining. (b-c) The protein and mRNA expressions of Iba-1 were detected using western blot and RT-qPCR. (d) The release of inflammatory cytokines was detected using ELISA. (e) The protein expressions of p-NF-KB p65 and P-IKβ were measured using western blot. *** *p* < 0.001 vs BV2, ^###^*p* < 0.001 vs HG (25 mM) + C3G 10umol/l

To elucidate whether the mechanism of C3G in inflammation was related to microglial activation, the effects of C3G on the proliferation and inflammation in HG-induced BV2 cells were comprehensively investigated. As Figure 3a depicted, the number of Iba-positive cells in HG group was significantly higher than that in normal BV2 cells and MG group, whereas the number was markedly reduced in HG (25 mM) + C3G 10umol/l group. Likewise, HG induction greatly upregulated the mRNA and protein expressions of Iba-1, which were then downregulated by C3G administration (p < 0.001) (Figure 3b-c). The expressions of pro-inflammatory cytokines TNF-α, IL-β and IL-6 and inflammation-related mediators exhibited a remarkable elevation in HG group, which was partially abrogated upon C3G administration (p < 0.001) (Figure 3d-e).

**Figure 4.**
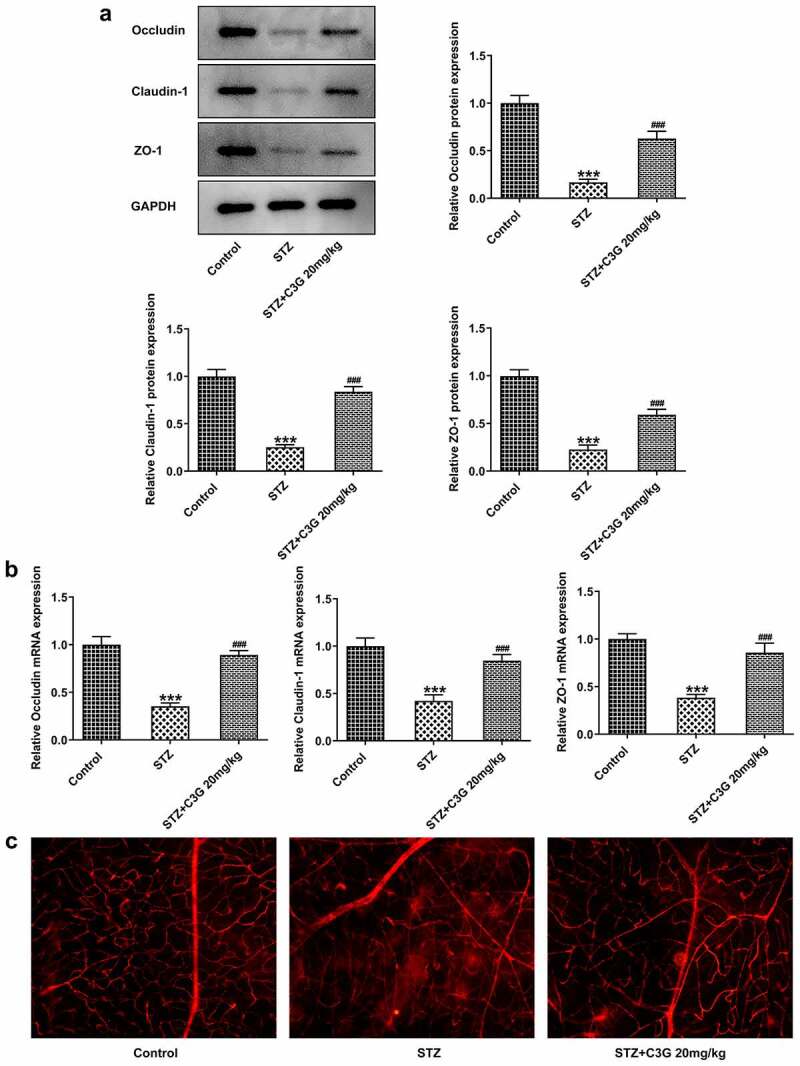
The effects of C3G on vessel leakage in the retinas of NPDR mice. (a-b) The protein and mRNA expressions of occludin-1, claudin-1 and ZO-1 were detected using western blot and RT-qPCR. (c) Evans blue permeation assay was used to detected vessel leakage. *** *p* < 0.001 vs Control, ^###^*p* < 0.001 vs STZ

**Figure 5.**
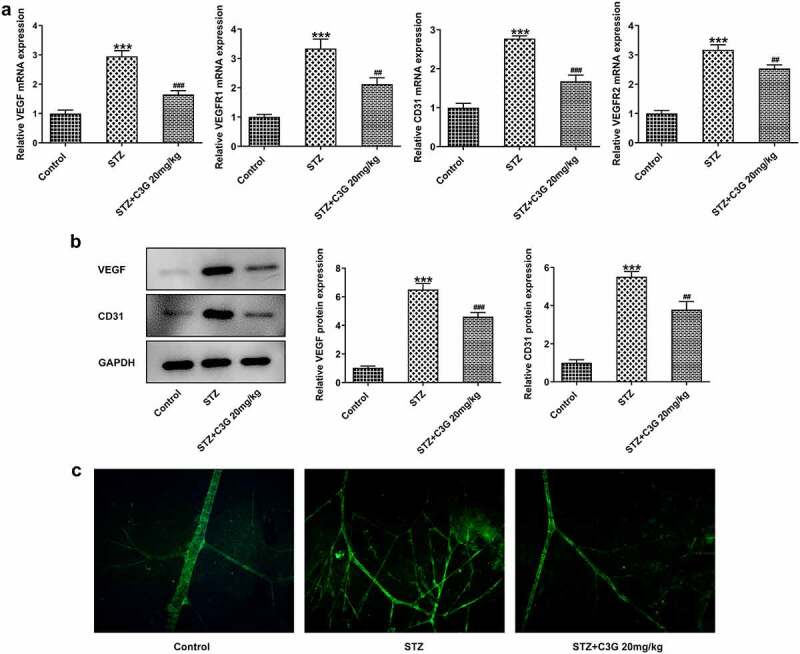
The effects of C3G angiogenesis in PDR mice. (a) The mRNA expressions of VEGF, VEGFR1, VEGFR2, and CD31 in the retinas of PDR mice were detected by RT-qPCR. (b) The protein expressions of VEGF and CD31 in the retinas of PDR mice were detected by western blot. (c) The expression of CD31 in the retinas of PDR mice was detected by immunofluorescent staining. *** *p* < 0.001 vs Control, ^##^ *p* < 0.01, ^###^*p* < 0.001 vs STZ

The effects of C3G on vessel leakage in the retinas of NPDR mice and angiogenesis in PDR mice Then, we sought to observe if there were any changes in vessel leakage of NPDR mice. Notably, sharp reduction of tight junction proteins such as occludin-1, claudin-1, and ZO-1 was observed in STZ group, by contrast with control group (p < 0.01) (Figure 4a). Nevertheless, C3G administration then significantly increased the expressions of these proteins. Evans blue showed that vessel leakage was significantly increased in STZ-treated mice, whereas CSG notably suppressed such leakage in STZ+C3G20mg/kg group (p < 0.01) (Figure 4b). Furthermore, results from RT-qPCR, western blot and immunofluorescent staining showed that angiogenesis was tremendously increased by STZ, while the promotive effects of STZ were partially abolished upon C3G treatment (p < 0.01) (Figure 5a-c).

**Figure 6.**
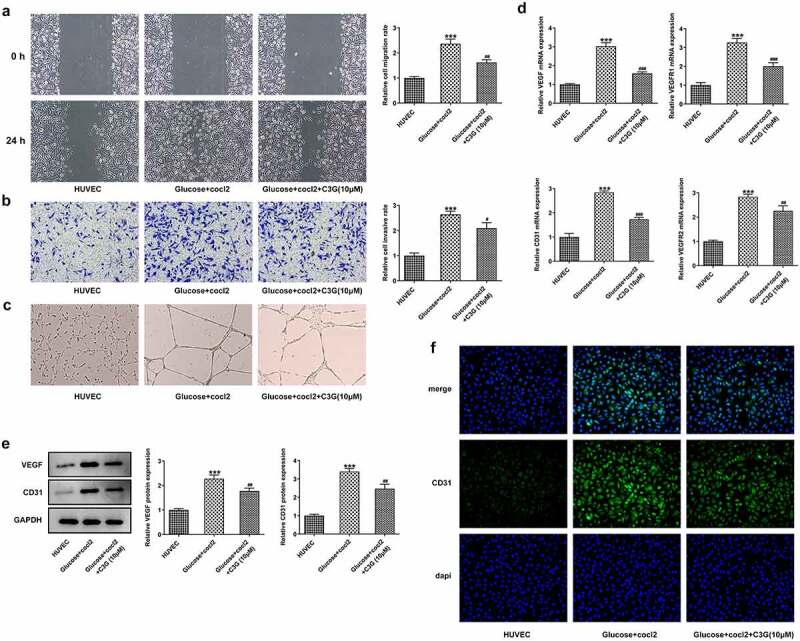
The effects of C3G on the migration, invasion and angiogenesis of in vitro DR model. (a) The migration was detected using wound healing. (b) The invasiveness was detected using Transwell. (c) The tube formation ability was detected using tube formation assay. (d) The mRNA expressions of VEGF, VEGFR1, VEGFR2, and CD31 were detected by RT-qPCR. (e) The protein expressions of VEGF and CD31 were detected by western blot. (f) The expression of CD31 was detected by immunofluorescent staining. *** *p* < 0.001 vs HUVEC, ^#^ *p* < 0.05, ^##^ *p* < 0.01, ^###^*p* < 0.001 vs glucose+cocl2

The effects of C3G on the migration, invasion and angiogenesis of in vitro DR model The duration of diabetes and the severity of hyperglycemia play important roles in DR [23], To further reveal the mechanism of C3G in angiogenesis, we simulated DR environment with HG and hypoxia by inducing HRECs with D-glucose (30 mM) and CoCl_2_ (200✓m/l) for 48 h. Obviously, the migration and invasion abilities of HRECs cells were remarkably promoted by glucose and cocl2, while C3G treatment partially abolished their promotive effects, evidenced by the decreased migration and invasiveness (p < 0.01) (Figure 6a, b). Meanwhile, the elevated tube formation ability of HRECs induced by glucose and cocl2 was suppressed after C3G pretreatment (Figure 6c). Regarding the angiogenesis of HRECs, the expressions of VEGF, VEGFR1, VEGFR2, and cluster of differentiation 31 (CD31) were significantly enhanced in glucose and cocl2-treat HRECs cells, which were then significantly inhibited by C3G administration (p < 0.01) (Figure 6d-f).

## Discussion

The anti-inflammation activity of C3G can attenuate diverse inflammation-related diseases. C3G exerts protective effects on acute lung injury rats via the inhibition of NF-✓B signaling pathway [21]. In addition, C3G can inhibit the activation of microglia and the production of inflammatory cytokines in LPS-induced BV2 cells, making it to be a promising therapeutic drug for the prevention and regimens of neurodegenerative diseases, of which hallmarks are neuroinflammation [24]. As retinal inflammation was an important marker in DR progression [25], the changes of pro-inflammatory cytokines in DR model in vivo were also detected upon C3G exposure. Consistent with the above-mentioned evidence, the present study indicated that the contents of pro-inflammatory cytokines including TNF- alpha, IL-1 beta, and IL-6 and inflammation-related markers were reduced upon C3G administration, suggesting the anti-inflammation function of C3G in DR model in vivo. Higher levels of TNF-alpha, IL-1beta, and IL-6 were found in the serum of type 2 diabetes mellitus compared with healthy control, which correlate with the development and progression of DR [26–28]. Moreover, there are certain association between increased aqueous concentrations of cytokines and the severity of patients’ diabetic retinopathy [29]. Some reporters demonstrate that the expression of TNF-alpha, IL-1beta, and IL-6 are closed associated with the modulation of NF-kappaB signaling pathway in vivo and vitro model of DR [30–32].

Intriguingly, microglial activation, neurotoxicity and tissue damage in the diabetic retina have been linked together according to the current evidence that has validated the significance of inflammation in DR progression, during which microglia are resident immune cells in the retinas [33]. Here, decreased expression of Iba-1 in STZ+C3G20mg/kg group reflected the impacts of C3G on obstructing microglial activation, which was consistent with previous findings, that are, C3G could protect neurons from injury by mediating microglia activation [24]. Interestingly, the inflammatory responses in STZ-induced mice model and DR cell model in vitro could be alleviated by C3G. Overactive microglia can release a large amount of pro-inflammatory cytokines [34], which may illustrate that the high production of pro-inflammatory cytokines is the final outcome of STZ induction.

Microglial cells, which are gatekeepers that safeguard the central nervous system from damage, involve a series of changes including proliferation, immunoreaction, and migration through their activation [35,36]. In this paper, the invasion, migration, and tube formation of HRECs stimulated under HG conditions were restrained by C3G administration. A previous study indicating that enhanced levels of VEGFR1 and VEGFR2 promoted cell proliferation, migration, and tube formation processes has instructed us to further investigate the neovascularization of DR model upon C3G exposure [37].

Since retinal vascular leakage is common to see in DR patients, the state of vascular leakage was measured in NPDR mice by determining the expressions of tight junction proteins, including occludin-1, claudin-1, and ZO-1 which are closely linked to vascular permeability in diabetes [38,39]. We found that the decreased expressions of tight junction proteins caused by STZ were significantly reversed after C3G treatment, proving that C3G plays an essential role in STZ-induced retinal vascular leakage which occurs in the early stage of DR. The typical vessel-related property of DR has supported the occurrence of accumulating evidences that note the critical role of proangiogenic factors such as vascular endothelial growth factor (VEGF) in the leakage occurring in the advanced stage of retinopathy [38]. VEGF, which contributed to the increased leakage through retinal vascular walls, was found to be overexpressed in DR rodent models and vitreous of human patients [40,41]. Consistently, the angiogenesis of HUVECs induced by glucose and cocl_2_ was promoted when the expression of VEGF was elevated. However, C3G treatment potently suppressed the angiogenesis in these cells. CD31 was a cell adhesion molecule involved in angiogenesis or the formation of new vessels [42]. CD31 staining showed a decreased number of new vessels upon C3G treatment, suggesting the repressive role of C3G in neovascularization. However, which pathway C3G follows to produce desired pharmacological action still needs further exploration. There was a study demonstrated that the potential protective role of C3G could be associated with SirT1/NF-κB signaling pathway, miR-204-5p/SIRT1, or AMPK/mTOR pathway [43–45]. In DR, detailed information about the retinal changes detected by histomorphological assessment on the retina, or fundus photograph, or optical coherence tomography is lacking for evaluating retina function, which is the limit of this study, which will be taken consideration in future studies. Additionally, the effects of C3G on blood glucose and weight gain, as well as its mechanism in reducing vascular leakage and neovascularization require further studies.

## Conclusion

To sum up, the results outlined in this study, which involved the in vitro and in vivo DR models, provided more lines of evidence for the efficacy of C3G in DR regimens. By confirming the role of C3G in inhibiting vascular leakage regulated by microglia activation in early DR and angiogenesis in advanced DR, this study pointed out the potential of C3G as a therapeutic drug for DR management.

## Data Availability

The datasets used and/or analyzed during the current study are available from the corresponding author on reasonable request.

## Highlights

1. C3G produces a potential inhibitory effect on the vascular leakage in NPDR.

2. C3G is able to reduce neovascularization in PDR.

3. C3G exerts anti-inflammatory effects via modulating NF-κB pathway and activation of microglial cells.

4. C3G inhibits invasion, migration and tube formation of HRECs stimulated by high glucose.
